# Eculizumab for paroxysmal nocturnal haemoglobinuria: catastrophic health expenditure in Nepalese patients

**DOI:** 10.1186/s13023-023-02779-2

**Published:** 2023-06-30

**Authors:** Sugat Adhikari, Surendra Sapkota, Suraj Shrestha, Kshitiz Karki, Anjan Shrestha

**Affiliations:** 1 Shreegaun Primary Health Care Center, Dang, Nepal; 2 Department of Internal Medicine, Ascension Saint Agnes Hospital, Baltimore, MD USA; 3 Maharajgunj Medical College, Institute of Medicine, Kathmandu, Nepal; 4 Annapurna Neurological Institute and Allied Sciences, Kathmandu, Nepal; 5 Department of Hematology, Tribhuvan University Teaching Hospital, Kathmandu, Nepal

**Keywords:** Catastrophic health expenditure, Eculizumab, Low and middle-income countries, Orphan drug, Paroxysmal nocturnal hemoglobinuria, Nepal

## Abstract

Paroxysmal nocturnal hemoglobinuria (PNH) results from a mutation in the phosphatidylinositol glycan class-A gene which causes uncontrolled complement activation with resultant intravascular hemolysis and its sequelae. Eculizumab is a terminal complement inhibitor that blocks this complement activation and has revolutionized the treatment of PNH but comes with an enormous price which can have catastrophic health expenditure in low-middle income countries (LMIC) like Nepal. Here, we discuss the potential way forwards in the treatment of PNH in Nepal and other LMICs.

**Supplementary Information:**

The online version contains supplementary material available at 10.1186/s13023-023-02779-2.

## Letter to the editor

Paroxysmal nocturnal hemoglobinuria (PNH), since its first definitive description in 1882 by the German physician Paul Strübing, has fascinated hematologists [1]. PNH results from a clonal proliferation of hematopoietic stem cells with a mutation in the phosphatidylinositol glycan class A (PIG-A) gene that results in the absence of two glycosylphosphatidylinositol (GPI) anchored proteins, CD55 and CD59. This causes uncontrolled activation of the complement system leading to excessive or persistent intravascular hemolysis causing anemia, hemoglobinuria, and complications related to the presence of plasma-free hemoglobin, including thrombosis, abdominal pain, dysphagia, erectile dysfunction, and pulmonary hypertension [2, 3]. The development of the terminal complement inhibitor eculizumab has revolutionized the treatment of PNH and, in turn, has revealed insights into the pathophysiology of the disease. Eculizumab is a humanized monoclonal antibody that blocks terminal complement activation by binding to the C5 complement and preventing it from being cleaved, thus preventing terminal complement activation [4]. Eculizumab is a lifesaving therapy that is associated with a greater than 50% reduction in transfusion requirements and a close to 70% reduction in the risk of thrombotic events and significant adverse vascular complications [5].

PNH is rare, with an incidence of 15.9 individuals per million worldwide [6]. Unfortunately, there is no available data on its incidence and/or prevalence in Nepal. On a PubMed search, only four cases of PNH have been reported and published in Nepal. The first case of PNH was reported in 2005 in Nepal. A 29-year-old Nepalese male, a manual laborer in Saudi Arabia presented to Patan Hospital with easy fatigability, pallor, and a few episodes of gum and nose bleeding for a year, initially misdiagnosed as megaloblastic anemia. Treatment was not described as the patient was referred to India where immunophenotyping was performed and diagnosed with PNH [7]. The second case reported in 2016 was of a 45-year-old woman with a known case of PNH for 5 years, who presented with severe headache, multiple episodes of vomiting, and confusion for 5 days along with weakness of the right side of the body and was found to have left transverse sinus thrombosis [8]. The other two cases were reported in 2021. One of them was a 26-year-old male from rural Nepal presenting with complaints of abdominal pain, fatigue and icterus who was inappropriately treated for four years prior to that presentation. He was found to have multiple unexplained thrombosis and a diagnosis of PNH was confirmed with a flow cytometry [9]. The other was a 38-year-old male, known case of PNH for 2 years, presented with left lower leg numbness, coldness along with loss of movement and was found to have popliteal artery occlusion, likely a complication of PNH, ultimately needing knee amputation [10]. PNH is often misdiagnosed with different types of anemia: iron deficiency, hemolytic, megaloblastic or refractory, and sometimes as myelodysplastic syndromes [7, 9]. Dr. Acharya et al. reported a case of a 28-year-old Nepalese woman with Herlyn Werner Wunderlich syndrome who presented with an ischemic cerebrovascular accident, pancytopenia, hemoglobinuria, and widespread abdominal thromboses suggestive of paroxysmal nocturnal hemoglobinuria [11] A PNH clone flow cytometry test was recommended, but due to the unavailability of the test in Nepal, a nationwide lockdown due to the coronavirus pandemic, and the poor financial situation of the patient, the test could not be performed [11]. This case is just the tip of the iceberg and vividly represents the plight of patients with PNH in less resourced nations like Nepal. The rarity of the disease, its association and similar presentation with other diseases; misdiagnosis of the disease condition, and limited funds may be some of the reasons for the absence of an epidemiological study of PNH in Nepal.

Eculizumab, the miracle drug for patients with PNH, unfortunately, comes with a huge price tag attached. Due to its status as an ‘orphan drug’ (a drug used to treat, prevent or diagnose a rare disease or condition), pharmaceutical companies have arbitrarily given it a very high price tag. The standard dosing regimen for eculizumab is 600 mg/week for 4 weeks (induction); 900 mg one week later; and then 900 mg every 14 ± 2 days (maintenance) [12]. In Nepal, where the country imports most of these kinds of drugs from its neighboring country, a single vial of 300 mg of eculizumab costs 2 lakh rupees (US $1,600). The standard dosage schedule of this drug would cost an individual more than $100,000 per year. However, the total subsidy provided by the Nepalese government for the treatment of cancer, heart disease, and kidney/liver diseases is only 100,000 rupees, which is equivalent to about US $800 currently, a recent increment from previous 50,000 rupees (equivalent to US $400) [13]. There is a lack of literature on health insurance in the case of Nepal, and our country is struggling to expand coverage of health insurance. The treatment package is usually limited to essential health care services [14]. A citizen of a nation with a per capita income of $1,223 [15], who suffers from PNH will never even have the opportunity to fight against it. Most low and lower-middle-income countries do not have effective financial protection schemes and rely primarily on out-of-pocket (OOP) payments for health financing [13]. The financial burden that the family incurs due to the disease is measured in terms of catastrophic health expenditure (CHE). If out-of-pocket expenses for health care exceed a certain proportion (generally 40% of non-food expenditure) of a household’s income, the expenditure is considered catastrophic [16]. Catastrophic payments capture the extent to which households face large financial shocks due to health payments [17]. In a study by Thapa et al. across Nepal, a tenth of households, most of them below the poverty line, residing in rural areas, suffering from chronic diseases, faced a catastrophic health burden [16]. In Nepal, major non-communicable diseases such as diabetes, asthma, and heart disease are often associated with catastrophic spending in the poorest households [18]. Therefore, it is well understood that expenditure for rare drugs such as eculizumab can be catastrophic to the majority of the Nepalese population. Protecting the population against the financial risk associated with poor health is one of the fundamental functions of the health system [16]. Establishment of social health insurance and general tax-based prepayment mechanisms may be long-term solid solutions to these problems [17]. Countries should be encouraged to establish prepayment schemes for health financing, as there is strong evidence that the larger the proportion of prepayment, the smaller the proportion of households that will face catastrophic health spending [19]. Although these steps might help minimize the CHE associated with chronic illnesses, it will still take several years for a poor nation like Nepal to be able to get such life-saving rare drugs without out-of-pocket expenses for its citizens.

The patients we reported above received blood transfusions, iron therapy, folic acid, and steroids to reduce hemolysis and thrombotic episodes; those with thrombosis also received anticoagulation. Corticosteroids can improve hemoglobin levels and reduce hemolysis in some patients with PNH, but long-term toxicity and limited efficacy limit enthusiasm for these agents [2, 20]. None of the reported cases of PNH in Nepal were on eculizumab and this was attributed to inaccessibility and financial constraints [7–10]. In a personal communication, Robert A. Brodsky, MD, Director of Division of Hematology at Johns Hopkins, mentioned that he never had a problem getting the drug for a US citizen and expressed his frustration of not being able to get many potential life-saving drugs in less-resourced nations. Although we struggle to get eculizumab despite 15 years from the date of approval, well-resourced nations are looking for next-generation complement inhibitors such as pegcetacoplan, a C3 inhibitor administered subcutaneously, and danicopan, a complement factor D inhibitor administered orally [21]. Both drugs are currently on trial and eliminate the need for blood transfusion by blocking intravascular and extravascular hemolysis [21, 22]. Another promising drug, ravulizumab, a C5 inhibitor, has been considered as a less costly and non-inferior alternative to eculizumab with a reduced dose frequency [23]. Allogeneic hematopoietic stem cell transplantation (HSCT) can possibly cure PNH. Although potentially curative, complications such as graft-versus-host disease, infection, organ dysfunction, lead to morbidity and mortality after HSCT [24]. It should only be offered as an initial therapy in countries where the drug is not available [2, 20]. Only limited cases of allogeneic HSCT have been performed in Nepal. The sharing of locally generated data could provide information on challenges and overall outcome in these patients.

In conclusion, despite revolutionizing the treatment of PNH with well-established efficacy and safety, eculizumab has not been established as the mainstay of treatment in Nepal’s PNH patients. Moving forward, the acquisition of the drug by the public system could be the best way to combat the overpricing of the drug [25]. This could still turn out to be quite expensive per patient for low- and middle-income countries like Nepal, and in this regard, manufacturers must resort to price reduction strategies for such countries. Similarly, more research is also currently required to discover new drugs that could be used as an alternative to eculizumab. Ensuring validated protocols for drug use, negotiating with pharmaceutical companies, and adjustment of the price based on gross domestic products by the government might be key steps to make the drug affordable [25].

## Electronic supplementary material

Below is the link to the electronic supplementary material.

Supplementary Material 1

## Data Availability

Not applicable.

## List of abbreviations

PNH: Paroxysmal nocturnal hemoglobinuria
GPI: glycosylphosphatidylinositol
CHE: catastrophic health expenditure
HSCT: hematopoietic stem cell transplantation
LMIC: Low-middle income countries
PIG-A: Phosphatidylinositol glycan class A
OOP: Out-of-pocket
