# Supplementary material for: Eculizumab for paroxysmal nocturnal haemoglobinuria: catastrophic health expenditure in Nepalese patients
Source: Orphanet J Rare Dis. 2023 Jun 30;18:172. doi: 10.1186/s13023-023-02779-2 (PMC10311788; doi:10.1186/s13023-023-02779-2)
Supplement: Supplementary file 1 — Supplementary Material 1 [file 13023_2023_2779_MOESM1_ESM.docx]

11-11-2022

To

The Editor,

Orphanet Journal of Rare Diseases.

We wish to submit a manuscript entitled “Eculizumab for Paroxysmal Nocturnal Hemoglobinuria: Catastrophic health expenditure in Nepalese patients” as a commentary for consideration by your prestigious journal.

In this article, we want to focus on the use of eculizumab - a highly effective drug for the management of Paroxysmal Nocturnal Hemoglobinuria (PNH). PNH is a rare disease with an incidence of about 15.9 individuals per million worldwide with grave complications.However, with the breakthrough of eculizumab, many patients worldwide have been successfully treated with the drug with more than 50% reduction in transfusion requirements for patients and close to 70% reduction in the risk of thrombotic events and significant adverse vascular complications. Despite all of these benefits, our primary concern is regarding the very high price to acquire the drug for a patient from a lower-middle income country like Nepal which makes the drug unaffordable for an average PNH patient in our country.

This study is significant because we have tried to review the few reported cases of PNH in Nepal and we have tried to open a conversation on behalf of all PNH patients in low-and-middle income countries like ours to ensure the affordability of this life saving drug.

We confirm that our work is original and has not been published elsewhere, nor is it currently under consideration for publication elsewhere.

We have no conflicts of interest to disclose.

Thank you in advance.

Sincerely,

Suraj Shrestha,

[multisurazz@gmail.com](mailto:multisurazz@gmail.com),

Maharajgunj Medical College,

Kathmandu, Nepal
